# Noninvasive Voiding Devices for Bedridden Women With Urinary Continence; Usability, Acceptability and Safety: A Scoping Review

**DOI:** 10.1155/nrp/2216719

**Published:** 2026-01-02

**Authors:** Ana Mesa La Guardia, Maria Teresa Prats Valls, Mónica Micó Cabedo, Pablo Juan Verdoy, Jaume Gual Ortí

**Affiliations:** ^1^ Teaching, Training and Research Unit, Alcoy Health Department, Alcoy, Spain; ^2^ Foundation for the Promotion of Health and Biomedical Research in the Valencia Region (FISABIO), Valencia, Spain; ^3^ Department of Materials and Industrial Technologies, University of Jaume I, Castelló, Spain; ^4^ Surgery Service, General d’Ontontinyent Hospital, Ontinyent, Spain; ^5^ Department of Mathematics, Universitat Jaume I, Av. Sos Baynat S/N, Castellón, 12071, Spain, uji.es; ^6^ Department of Industrial Systems Engineering and Design, Universitat Jaume I, Riu Sec Campus S/N, Castelló, 12071, Spain, uji.es

**Keywords:** acceptability, bedpans, bedridden women, catheterisation reduction, dignity, female urinals, female urination devices, macerator, patient safety, reprocessing, supine position, usability, washer–disinfector

## Abstract

**Background:**

Noninvasive female urination devices are widely used for bedridden women with urinary continence, yet concerns persist about usability, dignity and safety—especially in the supine position.

**Objective:**

To map and synthesise evidence on usability, acceptability and safety of noninvasive devices for supine female urination and to contextualise safety with handling/reprocessing practices.

**Methods:**

A scoping review following JBI and PRISMA‐ScR used a PCC framework and comprehensive searches (MEDLINE, CINAHL, Scopus, Embase, CUIDEN; Google Scholar/OpenGrey; March 2025), plus handsearching. Records were screened in duplicate; data were charted with a piloted form and narratively synthesised by device type and experience/safety domains.

**Results:**

Twenty‐one records met inclusion criteria: 17 core device‐focused studies and 4 contextual safety/handling sources. Core studies consistently reported discomfort, awkward posture, pain, embarrassment and dependence with traditional bedpans, alongside caregiver burden. Comparative evidence showed a clear preference for female urinals in eligible patients and greater acceptability for some alternative designs (e.g., disposable or inflatable variants); interventions that mobilised to the toilet or promoted respectful, skilled bedpan use were associated with reduced catheterisation. Safety findings indicated low adherence to education/reprocessing protocols and mixed signals regarding manual wiping versus automated disinfection. Contextual evidence documented persistent metal bedpan use in some health systems, wide variability in washer–disinfector/macerator availability and validation, and frequent manual emptying/rinsing—conditions linked to environmental contamination and antimicrobial‐resistant organism risk.

**Conclusions:**

Improving care for bedridden women requires a dual approach: (1) woman‐centred device redesign explicitly for supine use (fit, comfort, leakage control, dignity) with robust clinical validation and (2) system‐level implementation that minimises manual handling and assures validated, documented reprocessing (or fit‐for‐purpose disposable pathways), supported by staff training, zoning, PPE and auditing.

**Implications:**

Standardised outcome measures for comfort, dignity, leakage and contamination proxies, along with comparative effectiveness studies in supine populations, are needed to guide safe, dignified and sustainable practice.

## 1. Introduction

The management of urinary elimination in bedridden women who maintain continence presents a prevalent challenge within nursing practice. In hospital environments, particularly in acute care units or among postsurgical patients, it is common to encounter diagnoses such as self‐care deficits in toilet use, impaired physical mobility, decreased activity tolerance or difficulties in bed transfer and mobility [1]. These clinical diagnoses, as recognised by the North American Nursing Diagnosis Association (NANDA), accurately reflect the circumstances of many women who, despite retaining urinary continence, require assistance to access toilet facilities.

In such instances, devices like bedpans are frequently employed. However, the design of these devices has undergone minimal change since the 18th century. The traditional bedpan, characterised by its oblong shape, posterior opening and generally manufacturer in polypropylene, remains in widespread use without significant adaptation to the anatomical, functional and psychological needs of women [2, 3]. This situation highlights a concerning technological inertia that has not aligned with the advancements in person‐centred care. Beyond polypropylene, metal bedpans are still routinely used in several countries (e.g., Germany) due to durability; however, their rigidity and thermal properties may further compromise comfort and acceptance among female patients [4]. International and national surveys also show wide variability in materials (plastic vs. steel), reprocessing technologies (washer–disinfectors, macerators) and manual practices, underscoring the need to consider context when interpreting patient experience and safety [5, 6].

In addition to the physical discomfort described by patients—such as the coldness of the material, uncomfortable postures, associated pain and fear of leakage—the use of these devices can lead to complications such as skin lesions, urinary tract infections and unnecessary dependence on bladder catheterisation [7–9]. A negative impact on the perception of intimacy and dignity of female users has also been documented, especially when they share a room or other environmental factors increase emotional vulnerability [10].

Anatomical differences between men and women in the lower urinary tract should be considered when designing voiding devices. The female urethra measures 3 to 4 cm and is located in a region that demands precision in the placement of any collection system [7]. Despite this, most available devices have been designed with a neutral or male focus, which limits their efficacy and increases the risk of complications in bedridden women.

In addition, there are risks of cross‐contamination when the same device is used for urination and defecation, especially if proper cleaning is not ensured or if rigorous disinfection protocols are not followed [11, 12]. Studies such as that of Lepainteur et al. have shown that excreta management is a frequently neglected aspect of hospital infection control protocols, which focus almost exclusively on hand hygiene [11]. Consistently, the ISO 15883‐3 standard for human waste containers indicates that manual procedures should be avoided whenever possible, and multicountry surveys/presentations link poor bedpan management (manual emptying, spraying with cold water, lack of PPE) with environmental contamination and MDRO transmission risks [6, 13].

The scarcity of adapted solutions for continent but immobile women contrasts with the existence of devices for incontinent men, such as condom‐type external catheters [14]. Some innovative proposals have emerged in the field of female urination, but they have been mostly oriented to contexts of incontinence or use in an upright position [15, 16], which makes them of little use for women bedridden in the supine position.

The design of devices that respect the physiology, psychological needs and dignity of women is an unfinished business. As suggested by authors such as Tinnion and Jowitt, women‐centred solutions developed with a gender perspective can transform the caregiving experience [17]. In parallel, current evidence from nursing homes and hospitals shows that infrastructure and workflow (e.g., density/validation of bedpan washer–disinfectors, separation of clean/soiled zones, availability of lids/PPE) critically determine safety outcomes in bodily waste management, reinforcing the case for woman‐centred redesign aligned with robust reprocessing pathways [5].

Therefore, it is essential to explore the available evidence on the acceptability, usability and safety of existing noninvasive devices for bedridden women with urinary continence. This review is part of the ERGOMIC innovation project, aimed at developing a device specifically designed to allow urination in the supine position.

The objective is to investigate the available evidence on the usability and acceptability of noninvasive devices for female micturition in bedridden women with urinary continence.

## 2. Material and Methods

### 2.1. Design and Reporting Framework

We conducted a scoping review following the Joanna Briggs Institute (JBI) approach [18] and reported according to PRISMA‐ScR [19]. Eligibility and synthesis were structured using the Population–Concept–Context (PCC) framework.

• Population: adult women (≥ 18 years) with urinary continence who are bedridden (temporary or prolonged).
• Concept: noninvasive devices enabling female urination in the supine position (e.g., bedpans, urinals, funnels/collectors, near‐patient devices), with outcomes on usability, acceptability, comfort, dignity, efficacy (e.g., leakage) and safety (e.g., skin complications, contamination proxies).
• Context: hospitals (acute/postsurgical), long‐term/socio‐healthcare facilities and home care.

### 2.2. Research Questions

1. What are bedridden women’s perceptions of comfort, hygiene and dignity when using bedpans or other voiding devices?
2. How do the efficacy, comfort and user experience of alternative supine female micturition devices compare with traditional bedpans?

### 2.3. Eligibility Criteria

#### 2.3.1. Inclusion

• Women aged ≥ 18 years with urinary continence.
• Evaluation of bedpans, urinary collectors or other noninvasive devices enabling supine micturition.
• Study designs suitable for evidence mapping: qualitative, quantitative, mixed‐methods, clinical cases, reviews, quality‐improvement/pilot/feasibility studies, cost analyses and grey literature.
• No restrictions on language or publication year.

#### 2.3.2. Exclusion

• Studies focused exclusively on urinary or faecal incontinence.
• Devices designed exclusively for men.
• Studies without results related to experience, efficacy, safety, acceptability or dignity.

### 2.4. Information Sources

A comprehensive search was performed in March 2025 with the support of a specialist health sciences librarian. Databases: MEDLINE (PubMed), CINAHL, Scopus, Embase and CUIDEN. Grey literature was explored in Google Scholar (first 200 results by relevance) and OpenGrey. We also screened reference lists of included studies and relevant reviews (snowballing). Targeted handsearching and grey‐literature checks identified four contextual safety/handling sources addressing bedpan materials, reprocessing workflows and contamination/MDRO risk, which were included as a predefined contextual safety subgroup [4–6, 13].

### 2.5. Search Strategy

Search strings combined controlled vocabulary (MeSH/Emtree/CINAHL Headings) and free‐text terms. Beyond usability and acceptability terminology, we explicitly incorporated excreta management and reprocessing terms—as requested by the reviewer—to capture safety and handling conditions that may mediate women’s experiences.

Concept blocks (Boolean AND/OR):

• Device: “Bedpans”[Mesh] OR “Urination Aids”[Mesh] OR “Urine Collection Devices”[Mesh] OR Bedpan∗ OR urinal∗ OR “female urin∗” OR “voiding device∗” OR funnel∗ OR collector∗
• Experience/outcomes: “Comfort”[Mesh] OR acceptab∗ OR usability OR “patient experience” OR dignity OR efficac∗ OR leakage OR safety
• Handling/reprocessing/safety: washerdisinfector∗ OR macerator∗ OR “ISO 15883” OR reprocess∗ OR decontamination OR contamination OR “cross‐contamination” OR “Spaulding classification”

Example (PubMed):

 (“Bedpans”[Mesh] OR “Urination Aids”[Mesh] OR “Urine Collection Devices”[Mesh] OR Bedpan∗ OR urinal∗ OR “female urin∗” OR “voiding device∗” OR funnel∗ OR collector∗)
 AND (“Comfort”[Mesh] OR acceptab∗ OR usability OR “patient experience” OR dignity OR efficac∗ OR leakage OR safety)
 AND (washerdisinfector∗ OR macerator∗ OR “ISO 15883” OR reprocess∗ OR decontamination OR contamination OR “cross‐contamination” OR “Spaulding classification”)

**Figure 1:**
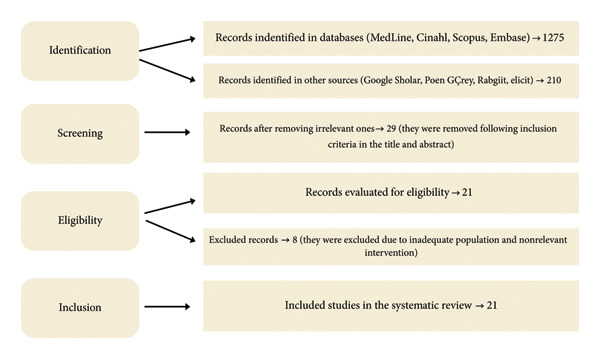
PRISMA‐ScR flow diagram (identification *n* = 1485; full‐text assessed *n* = 29; included *n* = 21).

Database‐specific adaptations are provided in Figure 1. No date or language limits were applied.

### 2.6. Study Selection

Records were deduplicated in Mendeley. Two reviewers independently screened titles/abstracts and subsequently full texts against eligibility criteria. Disagreements were resolved by discussion or by a third reviewer. Study selection was documented using a PRISMA‐ScR flow diagram (Figure 1).

### 2.7. Data Charting

A standardised data‐charting form was piloted on a sample of studies and refined iteratively. We extracted citation, country/setting, population, device description (material, geometry, positioning; single‐use vs. reusable), handling/reprocessing workflow (manual vs. washer–disinfector/macerator; availability of lids/PPE; clean/soiled zoning), design/methods, outcomes (comfort, acceptability, efficacy/leakage, safety/skin integrity, contamination proxies) and key findings. Charting was performed independently by two reviewers with consensus checks.

### 2.8. Synthesis of Results

Given heterogeneity in designs and outcomes, we conducted a narrative synthesis, grouping evidence by device type and by experience/safety domains (comfort/dignity; leakage/efficacy; skin complications; contamination/handling). Evidence that did not directly evaluate women’s usability but instead addressed mapping handling/reprocessing safety was synthesised as a contextual safety subgroup (*n* = 4 [4–6, 13]). We present descriptive summaries and evidence maps without meta‐analysis, consistent with JBI and PRISMA‐ScR guidance [18, 19].

### 2.9. Considerations on Bias and Evidence Completeness

• Selection and publication bias: multidatabase search; inclusion of grey literature; reference list screening; no date/language limits; dual‐reviewer screening to reduce subjectivity.
• Reporting bias: inclusion of theses/abstracts and quality‐improvement reports where they contributed relevant data.
• Study‐level limitations: as a scoping review, we did not conduct formal risk‐of‐bias scoring; we report design limitations (e.g., small samples, convenience sampling, lack of blinding) in the narrative synthesis [19].
• Context bias (per reviewer request): we proactively captured handling/reprocessing variables (manual emptying/rinsing, use/validation of washer–disinfectors/macerators, lids/PPE, clean‐soiled separation) and integrated a contextual safety subgroup [4–6, 13] to reflect safety signals that may mediate women’s experiences.

## 3. Results

### 3.1. Selection of Studies (Flowchart)

#### 3.1.1. Selection of Studies

A total of 1485 records were identified (databases = 1275; other sources = 210). After title/abstract screening, 29 articles proceeded to full‐text assessment. Eight full texts were excluded (noneligible population or intervention; insufficient outcome data), yielding 21 included records. Of these, 17 were core device‐focused studies (usability/acceptability/efficacy in bedridden continent women) and 4 were contextual safety/handling sources informing contamination and reprocessing risk. The process is detailed in Figure 1 (PRISMA‐ScR flow diagram).

#### 3.1.2. Characteristics of the Included Studies

The 21 records span 2000–2025 and encompass a range of designs: observational (*n* = 9), qualitative (*n* = 3), reviews/consensus or reports (*n* = 2), quality‐improvement projects (*n* = 2), technological innovation/validation (*n* = 2), mixed/other including grey literature (*n* = 1) plus the contextual safety/handling subgroup (*n* = 4; surveys and a conference abstract). Most studies were conducted in hospital settings; a smaller number involved home care or socio‐healthcare facilities. Main characteristics are summarised in Table 1 (*n* = 21).

**Table 1:** Main characteristics of the studies included in the review (*n* = 21).

Title of the study	Authors (Year)	Design	Main objective	Sample size (*n*)	Evaluated device	Main findings
Assessment of perceived patient comfort …	Cailleteau et al. (2024) [2]	Cross‐sectional observational	Assess patient comfort and bedpan management by caregivers	3007 participants (88% female), hospitals	Bedpan	83% reported physical discomfort. 59% reported difficulty in extraction. High physical burden for caregivers.
Patient experience with bedpans …	Gattinger et al. (2013) [7]	Quantitative descriptive	Describe the experience of patients with bedpans in acute care.	78 patients, acute hospital	Bedpan	Pain, uncomfortable posture, dampness, embarrassment, dependence.
Orthopaedic patients’ perceptions …	Cohen (2009) [8]	Qualitative	Explore perceptions of bedpans after orthopaedic surgery	10 patients post arthroplasty	Bedpan	Embarrassment, discomfort, perception influenced by nursing care.
Clients’ satisfaction with …	Kaur et al. (2020) [20]	Cross‐sectional observational	Measure satisfaction in hospital services	1614 patients, India	Bedpan	Mobility to the bathroom/toilets was a factor of dissatisfaction.
A project to increase nurses’ comfort …	McLain (2019) [21]	Quality improvement	Increase the use of bedpans in labouring women with epidurals	52 nurses, delivery unit	Bedpan/catheterisation	The use of bedpans increased from 5% to 19%. Decrease in long‐term urinary catheterisation.
How I became a bedpan superhero	McLain (2018) [22]	Clinical report	Describe strategies to promote the use of bedpans	N/A	Bedpan	Promoting respectful use. Influence of the professional approach.
Toilet use reduces catheterizations …	Hansen and Olsen (2015) [23]	Controlled trial	Compare mobilisation vs. use of bedpans in postoperative period.	152 patients, lumbar surgery	Bedpan/toilet	Less need for catheterisation in patients mobilised to the bathroom.
Report on continence product use	Murphy et al. (2023) [14]	Systematic review	Issue recommendations on continence products	International revision	Bedpans/urinals/absorbents	Recommended individual assessment, gender perspective and periodic review.
Female urinals for impaired mobility	Macaulay et al. (2006) [24]	Narrative review	Review female urinal options	N/A	Female urinals	Need to adapt the design to posture and urinary volume.
A guide to female urinals	McIntosh (2001) [25]	Grey literature	Assess availability of female urinals in the UK	N/A	Female urinals	Selection should consider posture, environment and practical needs.
Active urine collection device …	Tinnion and Jowitt (2000) [17]	Technological presentation	Introduce active device for disabled women	N/A	Active system	Design adapted to female needs. Evaluation pending.
Evaluation of excreta management …	Lepainteur et al. (2015) [11]	Multicentre survey	Evaluate excreta management in hospitals	536 units, France	Bedpan	Only 9% of personnel followed educational protocols. Contamination risk.
Reuse of hospital bedpans	Mineli et al. (2021) [12]	Comparative observational	Evaluate bedpan cleaning methods	180 bedpans, hospital	Bedpan	Manual cleaning with alcohol was more effective than automated cleaning.
Study of a new bladder device	Parreira et al. (2019) [26]	Quantitative cross‐sectional	Evaluate acceptance of inflatable bedpan vs. traditional bedpan	108 participants	Classic/inflatable bedpan	Increased acceptance of the inflatable bedpan. Problems with metal bedpans.
Patient’s ease of use …	Rodriguez and Levering (2016) [10]	Observational	Evaluate experience with female urinal	32 orthopaedic patients	Bedpan/female urinal	81.5% preferred female urinal. Reduction of catheterisation.
A disposable female urinal …	Thomas et al. (2024) [27]	Quality improvement	Evaluate usefulness of disposable device	103 patients + 118 professionals	Disposable urinal	Less pain, more dignity, less workload. 80% would recommend it.
Patient perceptions of bedpan usage	Schlachter (2015) [9]	Mixed	Explore perception of bedpan use	50 patients, hospital	Bedpan	Only 34% reported comfort. Common pain, insecurity, anxiety.
Global practices related to handling of faeces and urine in hospitals – results of an IFIC survey	Popp et al. (2014) [4]	International multicentre survey	Map hospital practices for urine/faeces management (materials, workflows, disinfection)	Hospitals across multiple countries (IFIC network)	Bedpans/urinals (reusable vs. single‐use; metal vs. plastic)	Persistent use of metal bedpans in some countries; differences in cleaning and disinfection implications for safety/contamination and perceived experience.
Surveys bedpan management in The Netherlands (1990 and 2010): progress in correct use of washer–disinfectors	Van Knippenberg‐Gordebeke (2011) [6]	National surveys (1990 vs. 2010)	Evaluate bedpan handling and correct use of washer–disinfectors	Hospital units in the Netherlands	Bedpans (focus on reprocessing with washer–disinfectors)	Improvement over time but ongoing incorrect/underuse of washer–disinfectors; frequent manual handling; risk of environmental contamination when reprocessing is unvalidated or bypassed.
P376: dirty bedpans and MDRO: partners in crime?	Van Knippenberg‐Gordebeke (2013) [13]	Conference abstract/poster (ICPIC)	Link inadequate bedpan handling with MDRO transmission	Hospital practice observations (summary of evidence and cases)	Bedpans (handling/reprocessing)	Manual emptying/rinsing and misuse of washer–disinfectors associated with contamination and MDRO risk; need to minimise manual handling and follow validated standards.
Bodily waste management and related hygiene practices in nursing homes of Vaud: a multicentre cross‐sectional survey	Glampedakis et al. (2025) [5]	Multicentre cross‐sectional survey	Describe excreta management and hygiene practices in nursing homes	Nursing homes in the canton of Vaud (Switzerland)	Bedpans/urinals; reprocessing circuits (manual, washer–disinfectors, macerators)	High between‐facility variability in materials and reprocessing; gaps in clean/soiled zoning, PPE use, and equipment validation; direct implications for perceived hygiene, safety, and workload.

*Note:* Summary of authors, design, objectives, population, type of device evaluated and main results related to usability, acceptability and safety.

### 3.2. Results of Individual Studies

#### 3.2.1. Patient and Caregiver Perception of Usability and Acceptability

Across core studies, traditional bedpans were consistently associated with coldness, uncomfortable posture, pain, embarrassment and loss of dignity [7–9]. More than 60% of participants reported negative experiences related to hygiene, posture and dependence on staff [8–10]. From caregivers’ perspectives, a large multicentre survey reported > 80% perceiving difficulty with complete evacuation and complex handling, with implications for musculoskeletal burden [2].

#### 3.2.2. Comparison Between Traditional Bedpans and Alternative Devices

Comparative studies found a clear preference for female urinals over bedpans in appropriate candidates [10, 20]. In Rodriguez and Levering [10], 81.5% of women rated the female urinal as more comfortable than the bedpan and 90.6% would recommend it. Disposable solutions (e.g., EasyWee™) were associated with less pain, greater dignity, reduced catheterisation and easier hygiene, with 80% of professionals recommending their use [27]. Patients also reported fluid restriction out of fear of using the bedpan [27]. Evidence from inflatable/alternative bedpans suggested higher acceptance versus classic/metal bedpans but highlighted practical issues that warrant design refinement [26].

#### 3.2.3. Impact on Catheterisation and Associated Complications

Inadequate bedpan use was linked to increased bladder catheterisation, with associated UTI risk. Educational and workflow interventions increased successful bedpan use postepidural and reduced Foley catheterisation [21, 22]. Allowing toilet voiding in selected postsurgical patients reduced intermittent catheterisation rates, supporting noninvasive, mobility‐oriented strategies when feasible [20].

#### 3.2.4. Opinion and Knowledge of Healthcare Personnel

Evidence indicates limited awareness of gender‐adapted alternatives among staff. Reports and reviews called for training on device selection considering posture, environment and volume, and noted variable efficacy across products (e.g., Spilpruf®, Verdaguer®, Bridge®), depending on positioning and mobility [14, 23]. This aligns with calls for woman‐centred redesign and evaluation in supine, bedridden users [17, 23].

#### 3.2.5. Safety and Hygiene in the Use of Devices

Core safety studies identified low adherence to reprocessing/education protocols [11] and reported findings on manual wiping with 70% alcohol versus automated disinfection that should be interpreted cautiously given methodological limitations [12].

Crucially, the contextual safety/handling subgroup documented wide variability in materials and workflows, including persistent use of metal bedpans, differences in washer–disinfector/macerator availability and manual emptying/rinsing practices, which are associated with environmental contamination and MDRO risk [4–6, 13]. These contextual data help explain users’ negative perceptions of hygiene, comfort and dignity and support minimising manual handling while ensuring validated, documented reprocessing pathways where reusable devices are employed [4–6, 13].

#### 3.2.6. Risk of Bias of Included Studies

Given the scoping design, a formal meta‐analytic appraisal was not undertaken; however, study‐level limitations were documented. Several core studies had moderate to high risk due to small/convenience samples, self‐report and lack of blinding or confounder control [7–9, 21, 27]. Innovation and grey‐literature items (e.g., 13, 21) were considered high risk in the absence of independent validation or comparative outcomes. By contrast, the controlled trial by Hansen and Olsen [20] and the empirical evaluation by Parreira et al. [26] showed better internal validity despite design constraints.

The contextual safety/handling sources [4–6, 13] were surveys/abstract not designed to assess usability outcomes; therefore, they were synthesised narratively as a predefined contextual subgroup and are flagged as such in the evidence tables rather than appraised with the same tools. Detailed risk‐of‐bias judgements and instruments used are presented in Table 2.

**Table 2:** Evaluation of the risk of bias in the articles identified.

Article	Scale	Risk of bias	Factors
Assessment of perceived patient comfort and ease of bedpan handling by caregivers, a cross‐sectional survey	Bias risk assessment with Newcastle–Ottawa scale (NOS)	Moderate risk of bias	Lack of adjustment for confounding factors and lack of comparability between subgroups.
Patient experience with bedpans in acute care: a cross‐sectional study	Bias risk assessment with Newcastle–Ottawa scale (NOS)	Moderate‐high risk of bias	Small convenience sample, lack of adjustment for confounding variables.
Orthopaedic patient’s perceptions of using a bedpan	Bias risk assessment with Newcastle–Ottawa scale (NOS)	High risk of bias	Small sample, lack of data triangulation and lack of adjustment for confounding factors.
Cross‐sectional study of clients’ satisfaction with outpatient and inpatient services of public health facilities of a North Indian state	Bias risk assessment with Newcastle–Ottawa scale (NOS)	Moderate risk of bias	Lack of adjustment for confounding factors, lack of multivariate analysis, lack of data triangulation.
A project to increase nurses’ comfort in offering bedpans to women laboring with epidural analgesia	ROBINS‐I risk of bias in nonrandomised studies of interventions	Moderate‐high risk of bias	Lack of adjustment for confounding, use of self‐reported measures, lack of blinding.
How I became a bedpan superhero	Critical appraisal skills programme (CASP)	High risk of bias	This is a personal reflection without structured methodology, lack of data collection, lack of systematic analysis and possible confirmation bias.
The number of in‐out catheterisations is reduced by mobilising the postoperative patient with bladder needs to the toilet in the recovery room	Cochrane risk of bias (RoB 2)	Moderate risk of bias	Possible observation bias due to lack of blinding and improvable randomisation method.
Management using continence products: Report of the 7th International Consultation on Incontinence	ROBIS (risk of bias In systematic reviews)	High risk of bias	The lack of meta‐analysis, absence of structured assessment of study quality, and declared conflicts of interest increase the risk of bias in this review.
Female urinals for women with impaired mobility	Descriptive product development study (adapted from STROBE and technology assessment criteria)	High risk of bias	This is an article of a divulgative and innovative presentation nature, without independent validation or quantitative data. It should be considered preliminary material, not robust evidence
A guide to female urinals	Adapted STROBE or adapted guides for educational articles.	High risk of bias	As an educational article without experimental data or objective comparison, it has an inherent bias of expert opinion.
The active urine collection device: a novel continence management system focussing particularly on the needs of disabled women	Technological innovation studies (based on adapted TIDE/STARD).	High risk of bias	In the absence of clinical outcomes and comparisons, the risk of bias is high. The article should be interpreted as preliminary innovation material.
Evaluation of excreta management in a large French multi‐hospital institution	Risk of bias assessment (adapted from STROBE for observational studies)	Moderate risk of bias	Although it is a robust, large‐scale study, it relies on self‐reporting, does not include direct observation, and does not assess clinical outcomes.
Reuse of hospital bedpans	Risk of bias assessment (adapted from STROBE and guidelines for comparative experimental studies)	Moderate risk of bias	It is a solid study, but with limitations in randomisation and without real microbiological validation. It should be interpreted with caution.
Study of the innovative characteristics of a new technology for bladder and intestinal elimination: An empirical study for the evaluation of ease of use and perceived utility	Risk of bias assessment (adapted from STROBE and guidelines for comparative experimental studies)	Moderate‐high risk of bias	Nonrandom sampling and absence of confounding control. Nevertheless, the methodological quality of the instrument, the robust statistical analyses and the consistency in the presentation of results reinforce its internal validity as an exploratory and preliminary study.
Patient’s ease of use, comfort, and satisfaction with the female urinal	Risk of bias assessment with the adapted STROBE tool and guidelines for experimental comparative studies	Moderate‐high risk of bias	Nonrandom sample, lack of validated instruments and lack of control of confounding variables.
A disposable female urinal bottle (the EasyWee tm pending) improves patient experience for immobilised females with lower limb fractures	STROBE risk of bias assessment and guidelines for experimental comparative studies	Moderate risk of bias	Absence of a structured control group. No formal validation of the instrument. Lack of confounding control.
Patient perceptions of bedpan usage and comfort levels	Assessment of risk of bias with adapted STROBE scale and guidelines for experimental comparative studies	Moderate‐high risk of bias	Nonrandom sample size and selection. Lack of validation of the collection instrument. Absence of confounding control
Global practices related to handling of faeces and urine in hospitals—results of an IFIC survey	JBI analytical cross‐sectional checklist/AXIS (survey)	Moderate	Multicountry self‐reported survey; sampling frame/response rates variably reported; heterogeneous measures across hospitals/countries; limited confounder control
Bodily waste management and related hygiene practices in nursing homes of Vaud: a multicentre cross‐sectional survey	JBI analytical cross‐sectional checklist/AXIS (survey)	Moderate	Facility‐level selection; self‐report; operational definitions mostly clear but on‐site validation of reprocessing not systematic; system‐level signals rather than clinical outcomes.
Surveys bedpan management in The Netherlands (1990 and 2010): progress in correct use of washer–disinfectors	JBI analytical cross‐sectional checklist/AXIS (two national surveys)	Moderate–High	Self‐reported practices; potential non‐response bias; limited comparability across survey years; correct use of washer–disinfectors not independently verified.
P376: Dirty bedpans and MDRO: partners in crime?	AACODS (grey literature/abstract: Authority, accuracy, coverage, Objectivity, date, significance)	High	Conference abstract with abbreviated methods; no detailed primary data or statistical analysis; useful as signal evidence, not for causal inference

The results of this review highlight several important themes regarding the current state of noninvasive voiding devices for bedridden women, which warrant further discussion.

## 4. Discussion

This scoping review shows that noninvasive voiding devices—especially traditional bedpans—remain widely used for bedridden continent women, despite consistent reports of discomfort, loss of dignity and dependence on staff [7–10]. Across core studies, patients described bedpans as cold, painful and awkward, with negative emotional impact that was more pronounced in women with reduced mobility [7–9]. From the caregiver perspective, a large multicentre survey documented complex handling and difficulty achieving complete evacuation, with implications for musculoskeletal burden and workflow [2].

Beyond usability, safety and hygiene emerged as critical, interlinked concerns. Core hospital evidence identified low adherence to education/reprocessing protocols [11] and conflicting signals about manual wiping vs automated disinfection that should be interpreted cautiously given methodological limitations [12]. Complementing these findings, the contextual safety/handling subgroup [4–6, 13] highlighted wide variability in materials and workflows—including the persistent use of metal bedpans in several systems, differences in washer–disinfector/macerator availability and validation, and frequent manual emptying/rinsing—all of which are associated with environmental contamination and MDRO risk [4–6, 13]. Together, these data suggest that women’s negative perceptions of hygiene, comfort and dignity are not merely product issues but also consequences of work‐as‐done conditions (manual handling, inadequate zoning, limited PPE, suboptimal validation of equipment).

Importantly, these safety signals align with international practice guidance for washer–disinfectors that emphasise minimising manual handling and ensuring validated, documented reprocessing pathways [28, 29]. Where reusable devices are retained, adherence to such pathways appears essential; alternatively, disposable or near‐patient solutions may mitigate some risks if fit‐for‐purpose and acceptable to users [22–25, 30]. At the same time, comparative device studies point to clear preferences for female urinals in selected patients [10, 27] and to improved acceptance with certain alternative designs (e.g., inflatable variants), albeit with practical issues that warrant further optimisation [26]. Interventions that mobilise to the toilet when feasible or that promote respectful, skilled use of bedpans have been associated with lower catheterisation rates, with potential downstream reduction in catheter‐associated harms [20, 21, 23].

A recurring cross‐cutting gap is staff training and awareness. Reviews and reports highlight limited familiarity with gender‐adapted alternatives and with context‐dependent selection (posture, environment, urinary volume), which constrains implementation [14, 23]. Moreover, much of the available technology has been developed from male or “neutral” design baselines, with insufficient attention to female anatomy and the supine context, perpetuating a historical shortfall in woman‐centred care [14, 17, 25]. Even where innovation is promising, clinical validation in real‐world supine use is inconsistent, and studies often rely on small, convenience samples with limited confounder control [17, 23, 26, 27].

Taken together, the evidence supports a dual strategy:

1. Device redesign explicitly centred on bedridden women—addressing anatomical fit, comfort (thermal/pressure), leakage control, dignity and ease of independent positioning—and evaluated with robust usability and safety endpoints in the supine position; and
2. System‐level implementation that reduces manual handling and strengthens reprocessing quality (validated washer–disinfector use or appropriate disposable pathways), supported by staff training, clean/soiled zone separation, PPE availability and routine auditing [8, 9, 23–25, 30].

Finally, this review also surfaces research needs: standardised, validated outcome measures for comfort, dignity and leakage; comparative trials that incorporate workflow and contamination proxies; and evaluations that weigh sustainability and cost alongside clinical effectiveness. Advancing a woman‐centred, safety‐aware approach is likely to improve patient experience, decrease unnecessary catheterisation and enhance occupational safety for caregivers.

## 5. Conclusions

The evidence indicates that currently available noninvasive female voiding devices have material limitations in usability, acceptability and safety. Traditional bedpans are consistently linked to discomfort, vulnerability, dependence on staff and perceived hygiene deficits among bedridden women [7–10]. While several alternatives show better comfort and dignity [10, 27] and are associated with reduced catheter use in specific pathways or after targeted interventions [20, 21], clinical validation in real‐world supine use and implementation quality remain uneven.

Safety signals cut across products and systems. Core hospital studies reported low adherence to reprocessing/education protocols and mixed findings on manual wiping vs. automated disinfection that require cautious interpretation [11, 12]. Complementing this, contextual safety/handling evidence documents wide variability in materials (including persistent metal bedpan use), reprocessing pathways (washer–disinfectors, macerators, manual handling) and work‐as‐done constraints that are associated with environmental contamination and MDRO risk [4–6, 13]. These conditions likely amplify women’s negative experiences and must be addressed alongside device redesign.

### 5.1. Implications for Practice

• Prioritise woman‐centred device selection and use in the supine position, favouring options with demonstrated comfort, dignity and leakage control [10, 26, 27].
• Where reusable devices are retained, minimise manual handling and ensure validated, documented reprocessing (e.g., correct washer–disinfector use and auditing), with clear clean/soiled zoning and PPE [8, 9, 23–25, 30].
• Implement education and competency‐based training so staff understand indications, positioning and device alternatives, reducing default catheterisation and suboptimal practices [14, 23].
• When feasible, embed mobility‐first pathways (e.g., assisted toilet voiding) to further reduce catheterisation and its downstream harms [20, 21].

### 5.2. Implications for Innovation and Policy

• Drive woman‐centred, evidence‐based innovation explicitly designed for supine micturition, addressing anatomical fit, thermal/pressure comfort, independent positioning and workflow integration.
• Evaluate new and existing devices with robust, standardised outcomes (comfort, dignity, leakage, reprocessing feasibility, contamination proxies) and comparative designs in the intended clinical context (bedridden, supine).
• Align procurement and policies with validated reprocessing (or fit‐for‐purpose disposable pathways) and monitor adherence to bias routine quality/safety audits [8, 9, 23–25, 30].
• Incorporate training, implementation support and data feedback into roll‐outs to avoid the know‐do gap and ensure sustainable adoption.

### 5.3. Research Priorities

• Develop and use validated measurement tools for comfort, dignity and leakage; include contamination and workflow metrics.
• Conduct comparative effectiveness studies in supine, bedridden populations, with adequate control of confounding and meaningful patient‐centred outcomes [26, 27].
• Examine system‐level determinants (staffing, zoning, washer–disinfector validation, macerator availability) that mediate safety and experience [4–6, 13].

In sum, improving care for bedridden women requires a dual approach: better devices designed for women in supine use and better systems that reduce manual handling and reliably ensure hygienic reprocessing. Together, these strategies can enhance comfort and dignity, lower unnecessary catheterisation and improve safety for patients and caregivers.

### 5.4. Relevance to Practice

• The text emphasises the limitations inherent in traditional devices for female micturition among bedridden women who maintain urinary continence, highlighting the necessity for significant advancements.
• The findings underscore the importance of prioritising patient comfort and dignity in the selection and utilisation of these devices. Furthermore, the text draws attention to the adverse effects that prolonged and inappropriate use of bedpans can have on both the physical and emotional well‐being of patients, as well as the physical demands placed on caregivers.
• There is evidence indicating a lack of training and confidence among healthcare personnel, which hinders the proper use of these devices and, consequently, affects the quality of care provided.
• It is advisable to invest in technological innovation to develop solutions that are safe, hygienic and anatomically appropriate, which could lead to a marked improvement in patient experience, a reduction in the unnecessary use of catheters and the creation of a safer and more humane hospital environment.

## 6. Limitations of the Study

This scoping review presents certain limitations that should be considered when interpreting its findings:

• Heterogeneity among the included studies: Studies with diverse methodologies, sample sizes and clinical contexts were identified, thereby complicating the generalisation of findings.
• Variable methodological quality: Several included studies exhibited moderate to high risks of bias, particularly in qualitative studies, grey literature or unvalidated innovation presentations [17, 24].
• Lack of meta‐analysis: Due to the scoping nature of this review, coupled with the heterogeneity of the studies, it was not possible to perform a quantitative synthesis.
• Limited representation of patients: The majority of studies focused on acute hospital settings, with limited representation of other environments such as home care or sociosanitary residences.
• Insufficient gender perspective: Although efforts were made to identify evidence specifically concerning bedridden women, the available studies seldom explicitly considered sex or gender differences in the design or implementation of the devices.

Despite these limitations, the review provides an up‐to‐date and valuable overview of the use, perception and challenges associated with female voiding devices in bedridden women.

## 7. Implications for Future Research

• It is necessary to develop and validate devices specifically tailored for bedridden women with preserved urinary continence, incorporating criteria of usability, hygiene, ergonomics and dignity.
• It is recommended to promote controlled clinical studies comparing traditional devices with innovative technologies from a gender perspective.
• The exploration of educational tools aimed at healthcare personnel is essential to enhance training, confidence and competence in the utilisation of alternative devices.
• Future research should also consider the organisational, economic and environmental impacts of employing well‐designed noninvasive devices.
• Finally, it is pertinent to integrate the patients’ perspectives in the processes of design, evaluation and selection of devices, adhering to participatory, person‐centred approaches.

## Conflicts of Interest

The authors declare no conflicts of interest.

## Author Contributions

Conceptualisation: Ana Mesa La Guardia, Maria Teresa Prats Valls, Mónica Micó Cabedo and Jaume Gual Ortí. Data curation: Ana Mesa La Guardia and Pablo Juan Verdoy. Formal analysis: Ana Mesa La Guardia and Jaume Gual Ortí. Funding acquisition: Ana Mesa La Gaurdia, Maria Teresa Prats Valls, Mónica Micó Cabedo and Jaume Gual Ortí. Investigation and methodology: Ana Mesa La Guardia. Project administrative: Pablo Juan Verdoy and Jaume Gual Ortí. Resources: Jaume Gual Ortí. Supervision: Pablo Juan Verdoy and Jaume Gual Ortí. Validation: Ana Mesa La Guardia, Pablo Juan Verdoy and Jaume Gual Ortí. Visualisation: Ana Mesa La Guardia, Pablo Juan Verdoy and Jaume Gual Ortí. Writing–original draft: Ana Mesa La Guardia, Maria Teresa Prats Valla and Mónica Micó Cabedo. Writing–review and editing: Ana Mesa La Guardia, Pablo Juan Verdoy and Jaume Gual Ortí.

## Funding

This study was supported by the LABORA‐LABS Impuls Programme (Universitat Jaume I, 2024).

## Data Availability

The data that support the findings of this study are available upon request from the corresponding author. The data are not publicly available due to privacy or ethical restrictions.
